# Stuck in a Rut of Thought—That Is Just a Barrier: Dysfunctional Metacognitive Beliefs, Limitation on Individual Freedom and Well-Being of Adolescents during COVID-19 Lockdown

**DOI:** 10.3390/ijerph20065151

**Published:** 2023-03-15

**Authors:** Natalia Kajka, Hanna Karakuła-Juchnowicz, Agnieszka Kulik, Paweł Szewczyk, Konrad Hryniewicz

**Affiliations:** 1 1st Department of Psychiatry, Psychotherapy and Early Intervention, Medical University of Lublin, 1 Gluska St., 20-439 Lublin, Poland; 2 The Department of Psychotherapy and Health Psychology, The John Paul II Catholic University of Lublin, Al. Racławickie 14, 20-950 Lublin, Poland; 3 Department of Marketing and Quantitative Methods, Faculty of Management and Quality Science, Gdynia Maritime University, 81-87 Morska St., 81-225 Gdynia, Poland

**Keywords:** metacognitive beliefs, isolation, well-being in the COVID-19 pandemic, adolescent, depression

## Abstract

Background: The aim of the cross-sectional study was to conduct an exploratory analysis of identifying factors related to mood, metacognitive beliefs, and limitation on individual freedom associated with lockdown restrictions during COVID-19, and to determine whether they may be relevant to the deteriorating well-being of adolescents. Methods: A total of 387 adolescents (M = 15.37; SD = 1.62): 85 with depression (DG) and 302 without any psychiatric diagnosis group (WPDG) were examined using the health survey and the CDI-2 questionnaire to assess the symptoms and severity of depression and MCQ-A to measure the intensity of dysfunctional metacognitive beliefs. Results: The feeling of restriction of freedom had an influence on worsened well-being in the whole group of responders OR = 4.15; *p* < 0.001 but was more in the DG than the WPDG (OR = 20.00; *p* < 0.001 vs. OR = 4.77; *p* < 0.001). Positive metacognitive beliefs were related to well-being (DG), but no effect was observed in the WPDG (OR = 0.88; *p* < 0.05 vs. OR = 1.05; *p* = 0.136). The lower age of the WPDG negatively impacted well-being (OR = 1.20; *p* < 0.05). Conclusions: Dysfunctional metacognitive beliefs and the feeling of restriction of freedom are important in the deterioration of adolescents’ well-being, but these factors have a stronger impact on well-being in the DG.

## 1. Introduction

### 1.1. Impact of the Pandemic on the Well-Being and Health of Young People

The COVID-19 pandemic is one of the greatest challenges of the 21st century. The disease that stems from the severe acute respiratory syndrome coronavirus (SARS-CoV-2) is considered a collective stressor. It is associated not only with the disease or death of the sick themselves or their relatives but also with chronic stress related to lifestyle changes, a sense of isolation, and depressed mood [1]. As in many countries, in Poland, measures have been taken to prevent the spread and contraction of COVID-19. In accordance with the regulation of the Ministry of Health, the lockdown in Poland lasted from March 2020 to May 2022 [2,3]. This situation forced huge changes in the daily functioning of both children and adults [4]. The biggest ones include the closure of schools, introduction of a curfew and transition to a remote learning system [5]. Parents, who often worked remotely at that time, had to reconcile the duties related to their profession and the education of their children in the next room. Young people had limited opportunities to move around and meet their peers. These disruptions to daily rhythms and tasks appear to have had a strong impact on adolescents’ well-being and mental health. Some researchers report that because of isolation, symptoms of secondary post-traumatic stress disorder could be identified in a quarter of children [6]. In addition, there is also an increase in the symptoms of depressive and anxiety disorders, an increased frequency of alcohol and cannabis use by this group of people [7,8,9]. These data indicate not only a rise in the severity of symptoms in adolescents who have previously struggled with mood disorders but also the emergence of symptoms in previously healthy children and young adults [6,7]. This is particularly worrying, since adolescence is the time when most mental disorders are revealed [10]. The peer group often provides important support for young people. The social sphere becomes a first mirror in which teenagers can see themselves against their peers and they develop a sense of group belonging. They develop their sense of independence and individuality from their parents as well.

### 1.2. Understanding Subjective Well-Being and Limitation on Individual Freedom

Unfortunately, changes in everyday life caused by restrictions on freedom, including transitioning to “online mode” may have had a particular impact on the well-being of teenagers [10,11]. So far, in studies, the subjective well-being of adolescents has been understood in two ways—the hedonistic and eudaimonic perspectives [12]. The first referred to global satisfaction with life and focused on general happiness and joy [12,13]. The second showed affective well-being—pleasant and unpleasant emotions experienced by adolescents [12]. According to the eudaimonic perspective, well-being occurs when an individual lives in accordance with their subjective beliefs and values. The situation of the pandemic as a strong, collective stressor has significantly affected the disruption of this construct in adolescents. Especially when some restrictions have been imposed on them due to the prevention of the pandemic in Poland and in the world. This is confirmed by preliminary research conducted by The Organization for Economic Co-operation and Development (OECD). In its report, which included 151 youth organizations from 72 countries, the OECD found that the top five most important consequences were the restriction of young people’s individual freedoms [14]. Young people most often mentioned difficulties in moving around. Macip & Yuguero [15] took up the topic of limitation on individual freedom in the pandemic and presented an interesting paradox of the consequences of control resulting from restrictions and freedom. These authors note that in the long run, a temporary loss of limitation on individual freedom can provide greater personal freedom. Nevertheless, there is a need for more research on this topic [15]. In particular, when it comes to the impact of these restrictions on young people’s beliefs, which may determine their well-being.

### 1.3. Dysfunctional Metacognitive Beliefs and Mental Health Disorders in Adolescents

According to the cognitive model and the “cognitive susceptibility hypothesis”, it is assumed that people prone to depression have dormant, characteristic thought patterns. They are activated by stressful life events, which certainly include the COVID-19 pandemic. How people think about this difficult event has a significant impact on the development of their mood disorders, anxiety, mental health problems, or disorders [16,17]. According to Wells and Matthews, the metacognitive model indicates that people’s perceived worry serves as a specific strategy for dealing with newly generated worries in their lives [18]. This, of course, leads to a vicious circle in which the trigger generates falsely positive metacognitive beliefs (“If I worry, I will be able to organize myself better/I will cope with the problem that worries me better”), it impacts the biggest emotional experience and entails more worrying than before. It is related to the negative consequences of this worrying (“My worrying is driving me crazy”). Current beliefs about worry bring about strategies for avoiding and suppressing your worrying thoughts. Consequently, this leads to an increase in the intensity of worrying. This condition often makes it difficult to discover that thoughts are usually harmless and manageable [18,19]. Being stuck in a rut of thought and worrying affects mental well-being. Such observations confirm the crucial role of beliefs about worry.

### 1.4. Purpose of Research

This cross-sectional study had two research goals: to conduct an exploratory analysis of factors that may be important for the deteriorating well-being of adolescents who had a psychiatric diagnosis of a depressive episode (Depression Group—DG) and without any psychiatric diagnosis (WPDG). The following were considered: metacognitive beliefs, limitation on individual freedom related to lockdown restrictions during the COVID-19 pandemic, emotional problems and problems with functioning resulting from depressive symptoms, and sociodemographic variables. The second objective was to construct a model explaining the subjective deterioration of the respondents’ well-being in both study groups during the pandemic. Due to the fact that the pandemic is a collective stressor, this study was exploratory in nature, and some of the variables described later in the work were not only quantitative but also qualitative in order to familiarize us with the specificity of the impact of limiting freedom resulting from restrictions related to the pandemic on youth well-being.

## 2. Methods

### 2.1. Procedure

The study was conducted during the lockdown period in 2020–2021. Due to the prevailing epidemiological situation and the pandemic restrictions, the research was conducted by correspondence and contact. Participants from the WPDG were reached through social media, non-governmental organizations, and schools (contact with school psychologists). A link with the content of the poster inviting the participation in the scientific project was sent to school secretariats, on forums for parents and teachers, and on youth group websites. Parents who reported their child’s participation in the study by e-mail were scheduled for a telephone conversation with the project coordinator. Qualified participants (WPDG group)—meeting the inclusion criteria—were sent a link to the study. The content of the posters included in the announcement: “We are interested in how young people (13–18 years old) think. We invite volunteers together with their parents to complete the questionnaires”. Schools were closed at this time and students learned only remotely. The researchers had no other way to reach the subjects.

The process of obtaining data from responders from DG was coordinated by psychiatrists and psychologists in health care facilities. They made a targeted selection taking into account the criteria of inclusion and exclusion from the study, they established contact with parents and adolescents, and then they conducted the study.

The inclusion criteria for the WPDG were:

1. Written informed and voluntary consent of parents of participants and of study participants * to:
  (a) Take part in the study;
  (b) Process personal data (RODO) as part of this project (* according to Polish law, informed and voluntary consent to participate in the study is required from adolescents aged 16 and older).
2. Female and male adolescents;
3. Age between 12 to 18;
4. Adolescents who have never been diagnosed or treated psychiatrically.

The exclusion criteria for the WPDG were:

1. Lack of written informed consent to take part in the study or process personal data (RODO);
2. Age less than 12 or more than 18;
3. Adolescents who have a diagnosed mental disorder now or in the past and/or have been treated psychiatrically;
4. Attending a special school;
5. Hearing or vision impairment which makes it impossible to understand instructions and complete questionnaires.

The inclusion criteria for the DG were:

1. Written informed and voluntary consent of parents of participants * and of study participants to:
  (a) Take part in the study;
  (b) Process personal data (RODO) as part of this project (* according to Polish law, informed and voluntary consent to participate in the study is required from adolescents aged 16 and older).
2. Female and male adolescents;
3. Age 12–18;
4. Diagnosis of depressive episode (F.32) according to ICD-10 [20].

The exclusion criteria for the DG were:

1. Lack of written informed consent to take part in the study;
2. Age less than 12 and more than 18;
3. Attending a special school;
4. Hearing or vision impairment, which makes it impossible to understand instructions and complete questionnaires;
5. Occurrence of psychotic symptoms or other mental disorders.

The following research tools were used in the study:

1. Personal questionnaire completed by a child. It encompassed the questions regarding the impact of the pandemic on his/her limitation on individual freedom and well-being, age, gender and place of residence.
2. Children’s Depression Inventory 2 (CDI-2) by Kovacs et al. [21] examines the level of severity of depressive symptoms on two main scales: emotional problems and problems related to everyday functioning. The questionnaire consists of 28 items, the examination time is 10–15 min. Reliability for the general Cronbach’s α score is 0.84–0.87, and for the main scales 0.73–0.82. The tool makes a good distinction between healthy and depressed individuals.
3. The Metacognitive Beliefs Questionnaire (MCQ-A) for Adolescent Cartwright-Hatton et al. [18] in the Polish adaptation of Kajka & Kulik [22]. The MCQ-A examines the severity of five dysfunctional beliefs about how young people think about worry and fear. The questionnaire consists of 30 items assessed by young people on a scale of 1–4; the estimated time of the study is 10 min. Cronbach’s alpha reliability analysis for the adapted questionnaire was 0.874. The results for individual scales are comparable to the original version [18].

### 2.2. Characteristics of Study Group

The study involved 387 adolescents (M = 15.37; SD = 1.62), including 85 with depression (DG) (M = 15.38; SD = 1.61; 60% women) and 302 without any psychiatric diagnosis (WPDG) (M = 15.37; SD = 1.62; 59.6% women) (Table 1). Based on fulfilling all inclusion and none of the exclusion criteria, respondents with psychiatric diagnoses other than a depressive episode were not included in the study. Since patients with depression were qualified for this study from healthcare facilities (psychiatric hospitals, psychiatry clinics), the patients were provided with psychological therapy. Somatic illnesses such as allergies, diabetes, psoriasis, asthma, gastroesophageal reflux, and hypothyroidism were diagnosed in 17 (5.60%) adolescents from the WPDG and 18 (21.17%) from the DG and were treated with insulin, levothyroxine, inhaled glucocorticosteroids, and antihistamines. Additionally, participants from DG (N = 53; 62.40%) took antidepressants—SSRIs (selective serotonin reuptake inhibitor), tricyclic antidepressants, and anxiolytics.

### 2.3. Limitation on Individual Freedom

**Figure 1:**
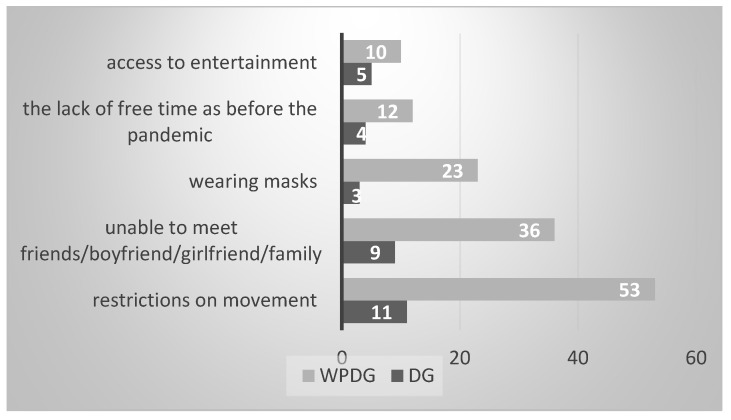
Examples of the most frequent limitation on individual freedom (N) described by adolescents.

Because the pandemic is associated with health and life risks, it is referred to as an extraordinary situation. Governments of all countries, protecting their citizens, introduced restrictions on the rights and freedoms of citizens. In this study, young people with depression and young people without a psychiatric diagnosis were asked if they felt limitations on individual freedom resulting from the restrictions related to the COVID-19 pandemic (Yes/No). They were then asked to describe how young people understand these limitations, taking into account their life situation. A total of 364 observations (people who specified how the restrictions affect the restriction of freedom) were included in the further analysis (N_WPDG_ = 286; N_DG_ = 78). The remaining persons did not note the impact of the limitation on individual freedom on their lives, or after the initial answer “yes”, they did not specify how. Detailed statistics are presented in Figure 1, in the DG, 30 people (38.46%) experienced limitation on individual freedom and in the WPDG—118 (41.25%). The most numerous complaints were restrictions on movement (N_DG_ = 11; 12.84%; N_WPDG_ = 53; 17.54%); inability to meet friends/boyfriend/girlfriend/family (N_DG_ = 9; 10.58%; N_WPDG_ = 36; 11.92%) and wearing masks (N_DG_ = 3; N_WPDG_ = 23; 7.61%). Less frequently, the respondents mentioned limitations related to the lack of free time as before the pandemic (N_DG_ = 4; 4.70%; N_WPDG_ = 12; 3.97%) and access to entertainment (going to the cinema, gym, or restaurant) (N_DG_ = 5; 5.38%; N_WPDG_ = 10; 3.31%). The analysis of the Chi2 test showed no significant differences between the DG and WPDG in the quantitative limitations mentioned above.

### 2.4. Well-Being of Adolescents

**Figure 2:**
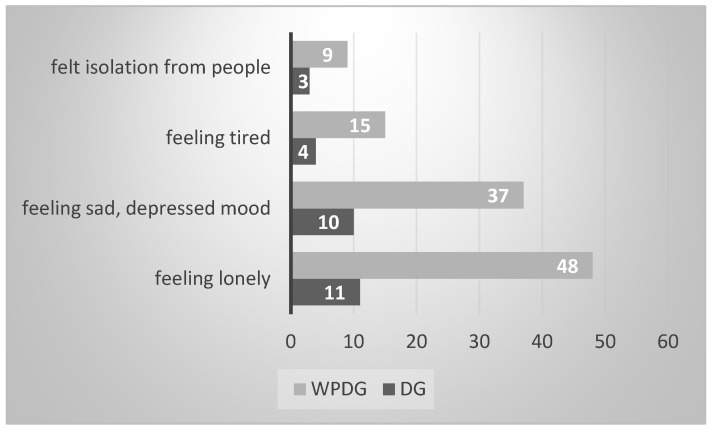
Examples of the most frequent feelings (N) described as deterioration in well-being adolescents.

In our project, adolescents were asked to assess their well-being which was understood as a change in their mental and physical state before and during the pandemic. The descriptive analysis of the results indicated a small percentage of respondents who improved their well-being (N_DG_ = 1; 1.1% vs. N_WPDG_ = 7; 2.64%). Detailed statistics are presented in Figure 2. It is noticeable that mainly, they emphasize such advantages as more free time and improved sleep. Unfortunately, most adolescents indicated deterioration in their well-being (N_DG_ = 60; 70.60% vs. N_WPDG_ = 166; 55%). From the qualitative analysis of the individual answers of all respondents, the most frequently mentioned were loneliness (N_DG_= 11; 12.94%; N_WPDG_ = 48; 15.89%), sadness and mood deterioration (N_DG_ = 10; 11.76%; N_WPDG_ = 37; 12.25%), fatigue (N_DG_ = 4; 4.70%; N_WPDG_ = 15; 4.96%), and isolation (N_DG_ = 3; 3.52%; N_WPDG_ = 9; 2.98%). The analysis of the Chi2 test showed no significant differences between the DG and WPDG in the quantitative limitations mentioned above.

### 2.5. Intensity of Depressive Symptoms among the Respondents

Due to the study of the well-being of adolescents with a diagnosis of depression, as well as young people without a psychiatric diagnosis, symptoms of depression measured with the CDI-2 scale are a controlled variable in this study. The analysis with the Student’s *t* test showed that there is a statistically significant difference between the groups in the severity of depressive symptoms (t(385) = 10.544; *p* = 0.001). The mean score on this scale is significantly higher in the DG (M = 26.44; SD = 9.94) than in the WPDG (M = 14.49; SD = 9.01). The obtained average results fall within the high (DG) and increased (WPDG) scores.

### 2.6. Intensity of Dysfunctional Metacognitive Beliefs

Table 2 presents both the results regarding the severity of dysfunctional metacognitive beliefs as well as the overall score regarding the severity of depressiveness. The conducted Student’s T Difference Tests among independent samples showed that the mean scores of individual scales in the DG group were different from each other in measures of metacognitive beliefs except for: Positive Metacognitive Beliefs (t = 1.682 (385); *p* = 0.093; M_DG_ = 10.68; SD_DG_ = 3.67; M_WPDG_ = 9.96, SD_WPDG_ = 3.44) and Cognitive Self-Awareness (t = 0.126 (385); *p* = 0.800; M_DG_ = 15.79; SD_DG_ = 3.05; M_WPDG_ = 15.74; SD_WPDG_ =3.25). The highest severity of dysfunctional metacognitive beliefs was observed in DG group as negative metacognitive beliefs (M = 17.60; SD = 4.43), while the WPDG group in terms of cognitive self-awareness, which allows us to estimate the selectiveness of teenagers’ attention (M = 15.74; SD= 3.35).

## 3. Data Analysis

All statistical analyses, tables, and figures were generated in R language [23] using “kableExtra” [24], and “jtools” packages [25].

### 3.1. Pre-Analysis Based on Propensity Score Matching Procedure

Due to the significant differences between the groups in terms of the place of residence, it was decided to remove some observations in order to reduce these differences. There was a possibility that in the circumstances of deterioration of well-being and limitation on individual freedom, the place of residence would be a confounding factor, because adolescents from the DG commonly lived in cities with more than 400,000 inhabitants (more social and physical restrictions during the pandemic), while the WPDG lived in rural areas (less social and physical restrictions during the pandemic). For this reason, a Propensity Score Matching procedure was used in order to balance significant differences between the groups only in terms of the place of residence. Metacognitive and depression scales were excluded from this procedure because they are inherently related to group membership. Propensity Score Matching is a statistical method that creates similarity between the groups in terms of balancing variables. The first step in this procedure is to calculate a balancing vector indicating the probability of belonging to group 1 and group 2 by logistic regression. The second step is to allocate observations that are similar to each other in terms of the value of the calculated balancing vector. Most often, observations from group 1 are assigned to group 2 using the nearest neighbor method (but there are other matching methods, e.g., strict similarity based on a list of the describing variables). This method makes it possible to assign observations from group 1 to observations from group 2 in terms of the balancing vector values. The “MatchIt” package was used to perform this procedure [26]. In the first step of the matching procedure, the place of residence was significantly related to group participation. Living in the cities with more than 400,000 inhabitants (vs. rural areas) was associated with a greater chance of being in a DG group OR = 2.02; *p* < 0.05; 95%CI (1.09–4.03). There were no associations in terms of residual levels of the place of residence variable. Before matching, the results are shown in Table A2 (Appendix A). In the matching procedure (second step), N = 34 participants were removed from the WPDG group because of the unbalanced vector values. This phase resulted in no differences between the groups in terms of the place of residence. Results are presented in Table A3 (Appendix A). All further analysis is based on balanced data.

### 3.2. Exploration Procedure

In order to explore the phenomenon of worsening of adolescents’ well-being in the data gathered, a logistic regression model was tested with main effects related to metacognitive, depression total score, and demographic variables. In order to indicate significant factors and interaction between them and the Group, a series of logistic regression models were tested. Significant main effects and significant interaction terms were included for further analysis (feeling of freedom × Group, and Group × Positive Metacognitive Beliefs, feeling of freedom, Positive Metacognitive Beliefs, Negative Metacognitive Beliefs, Cognitive confidence; Cognitive self-consciousness; superstition, punishment, and responsibility; Depressive Symptoms). All exploratory analyses are attached in Appendix A as series in Table A1.

## 4. Results

### The Results of the Multidimensional Model of Well-Being

The results of the final model (based on exploration results) are as follows. In Table 3, we present a tested multivariate model with interaction terms. Table 4 and Table 5 present, respectively, multivariate (multiple predictors) and univariate (single predictors) model estimates. Decisions related to testing the univariate model were based on the possibility of collinearity of predictors and the DG group’s small size. Differences between groups in terms of the OR estimates in both models were assessed by 95% confidence intervals.

**Figure 3:**
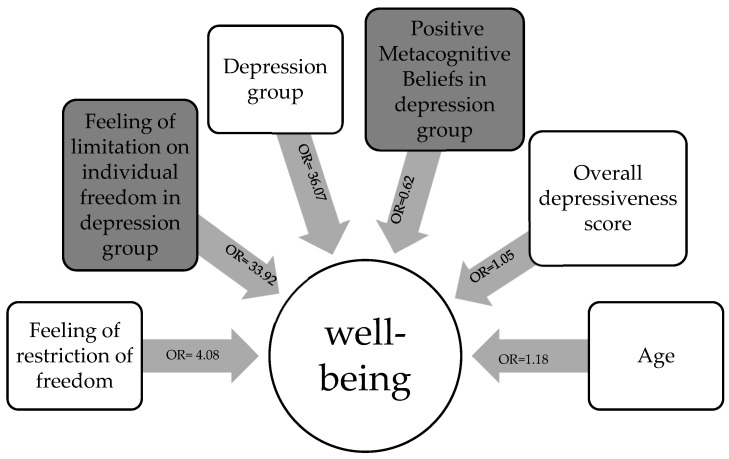
Graphical depiction of a multidimensional model of adolescents’ well-being during the COVID-19 lockdown.

For the reported model in Table 3, the Wald test showed that the tested model was significant F(9.377) = 8.46; *p* < 0.001 and explained around 32% of the state of well-being variability. The variable explained in this multidimensional model was well-being (1—deteriorating and 0—not deteriorating). The aim was to check which factors (meta-cognitive beliefs, depressive symptoms, limitation on individual freedom, age, gender, study group) would be significantly related to the deteriorating psycho-physical condition of adolescents. Results of the tested model (Table 3; Figure 3) show that a reduced feeling of freedom was related to a worsened state of well-being OR = 4.08; *p*< 0.001. A similar pattern was observed in age OR = 1.18; *p* < 0.042, Group OR = 36.07; *p* < 0.001, and in the interaction between the feeling of freedom and Group OR = 33.92; *p* < 0.01. There was also a significant interaction between Group and Positive Metacognitive Beliefs, but this pattern had opposite direction OR = 0.62; *p* < 0.01.

The simple effects of these interactions were tested and shown in Table 4 (multivariate model) and in Table 5 (univariate model). These results show that the reduced feeling of freedom had a higher influence on the worsened state of well-being in DG than WPDG OR = 169.12; *p* < 0.001; 95%CI (21.06–3823.00) vs. OR = 4.05; *p* < 0.001; 95%CI (2.40–7.08) (confidence intervals for these estimates did not overlap). The univariate models for both groups with only the feeling of freedom variable showed a similar pattern, but the difference between estimates was no longer significant OR = 20.00; *p* < 0.001; 95%CI (6.50–72.67) vs. OR = 4.72; *p* < 0.001; 95%CI (2.82–8.06) (confidence intervals for these estimates overlapped). Further analysis of simple effects for the Positive Metacognitive Beliefs showed that in the WPDG the Positive Metacognitive Beliefs were not related to the state of well-being OR = 1.01; *p* = 0.815; 95%CI (0.93–1.10), but, in the DG, an increased level of Positive Metacognitive Beliefs was related to better well-being OR = 0.65; *p* < 0.01; 95%CI (0.44–0.85) (confidence intervals for these estimates did not overlap). Again, the univariate regression model with only the MCQ-PMB variable showed quite similar differences OR = 1.06; *p* = 0.310; 95%CI (0.99–1.14) vs. OR = 0.88; *p* < 0.05; 95%CI (0.77–1.00) (confidence intervals for these estimates almost did not overlap). We also indicate that in the WPDG, increased age was related to better well-being OR = 1.22; *p* < 0.05; 95%CI (1.03–1.46), but in the DG this relation was insignificant OR = 1.00; *p* = 0.986; 95%CI (0.63–1.60). There was also another effect of Depression, but this effect was significant in the WPDG group OR = 1.06; *p* < 0.05; 95%CI (1.01–1.10). However, there was no effect in the DG group OR = 1.02; *p* > 0.05; 95%CI (0.93–1.1). Univariate analysis showed similar patterns in terms of estimates of Depression, and Age, but in the DG group Depression had a significant effect on well-being OR = 1.06; *p* < 0.05; 95%CI (1.01–1.12) (no effect was observed in WPDG group).

## 5. Discussion

The aim of this cross-sectional study was to identify factors that may be important for the deteriorating well-being of adolescents treated psychiatrically for depression (DG) and the group of adolescents never diagnosed psychiatrically (WPDG). The results obtained in this study indicate that selected metacognitive beliefs and limitation on individual freedom associated with the lockdown restrictions during the COVID-19 pandemic related to the well-being of adolescents in Poland. Young people who were treated for depression as well as those who had never been psychiatrically diagnosed or treated assessed that the restriction of their freedom had a significant impact on their well-being. The restrictions on movement, lack of possibility to meet important people, or wearing masks were the restrictions most frequently mentioned by the respondents. Findings from these studies overlap with the research on eudaimonic well-being. In this concept, autonomy also plays an important role, which would explain the relationship between the influence of the variables measured in this study [27].

The results of studies conducted on adults so far indicate that the pandemic and the challenges associated with it are related to dysfunctional metacognitive beliefs [28]. This manuscript along with several other publications complements this topic and addresses adolescent’s outcomes [22,29]. The results have shown that although in the tested single models the well-being of young people (DG and WPDG) is influenced by negative metacognitive beliefs or beliefs about superstitions, punishment and responsibility, the main dysfunctional metacognitive beliefs related to the well-being of depressed adolescents are positive beliefs about worrying. According to Papageorgiou & Wells [30], these beliefs are about ruminations. In the opinion of these researchers, it is a strategy characteristic of patients who want to cope with stress and symptoms of depression. Patients believe that if they think about their problem, they will find an answer to how to deal with the disease better. This gives them a false sense of control and locks them in a vicious circle of rumination about depressive symptoms [31]. This explains the results of this study, which indicates that the activation of this type of belief is related to the declaration of no negative impact of the pandemic on the well-being of young people with depression. That is why DG has, at the same time, a stronger sense of restriction of freedom due to the pandemic than the WPDG group. This suggests that the respondents from the DG were locked both in their illness (due to the vicious circle of rumination) and also had a limitation on individual freedom due to the restrictions resulting from the pandemic. Getting stuck in your worries and their possible causes and consequences is a harmful form of incompetent stress management [32]. This can lead to the development and persistence of mood disorders, which is a huge threat to young people struggling with previous difficulties. An in-depth analysis of this phenomenon, as well as primary depression prevention introduced to schools, can counteract the difficulties faced by young people, even once the pandemic ends [33]. These studies also show that a significant risk factor for the deteriorating well-being of WPDG adolescents is the younger age of the respondents. This may be related to the lower ability to meet their basic psychological needs in terms of school competence, social competence, or autonomy [34]. It is also noticed that younger children have a greater need for parental care and support than older youth [35]. Therefore, considering that this research was carried out at a time when Polish schools were closed and young people were learning online, the lack of parental availability and adapting to the new requirements could be more difficult for the younger respondents of this study.

### Limitations and Further Directions of Research

The restrictions on the freedom of movement and therefore the limitation of conducting research introduced during the pandemic resulted in a smaller than originally expected sample of patients diagnosed with a depressive episode and is not a representative random sample of the Polish adolescent population in terms of gender and demographics. This makes it difficult to transfer the obtained results to the population. Another limitation of this project was the lack of random sampling. In addition, although information was provided about the treatment and health status of adolescent people with DG, there is no detailed data on the type of therapy that they attended. Nevertheless, the presented results show significant and strong effects, which are an interesting premise for further analysis and further research. Clinical observation and results of many researchers all around the world have shown young people may still struggle with the effects of the pandemic for a long time [4,5,6,22]. Therefore, it seems important to correctly identify risk factors and the specific type and course of therapy undertaken by young people during that pandemic time. This would fill the gap in this paper. This kind of information will facilitate better planning of effective support.

## 6. Conclusions

Our cross-sectional and exploratory study pointed out that adolescents defined whether and how the limitation on their individual freedom during the lockdown affected their well-being. Due to the phenomenon of depressive reaction in adolescents (previously healthy), we were particularly interested in the similarities and differences between the group of adolescents never diagnosed psychiatrically and patients with an episode of depression. This study showed that dysfunctional metacognitive beliefs and the limitation on individual freedom are important in the deterioration of adolescents’ well-being, but these factors have a stronger impact on the well-being of individuals who had a psychiatric diagnosis of a depressive episode.

## Figures and Tables

**Table 1:** Sociodemographic characteristics of the respondents.

Sociodemographic Variables		DG		WPDG		*p*-Value
		N	%	N	%	
Gender	Male	34	40	122	40.4	0.947
	Female	51	60	180	59.6	
Place of residence	RA	21	25	92	30	0.036
	T 50	21	25	69	23	
	C 150	15	18	82	27	
	LC400	28	32.9	59	20	
limitation on individual freedom	Yes	30	38.42	118	41.25	0.656
	No	48	61.58	168	58.75	
Deterioration of well-being	Yes	60	70.6	166	55	0.01
	No	25	29.40	136	45	

Note: DG—depression group; WPDG—Without any psychiatric diagnosis group; RA—rural areas; T 50—Town with up to 50,000 inhabitants; C 150—City with up to 150,000 inhabitants; LC400—large cities with up to 400,000; *p*-value—Pearson’s Chi-squared test or U Mann–Whitney test was used as appropriate for the data.

**Table 2:** Descriptive characteristics of the results of MCQ-A and CDI-2 subscales.

Variables		DG	WPDG	*p*
		N = 85	N = 302	
MCQ-PMB	M	10.68	9.96	0.093
	SD	3.67	3.44	
MCQ-NMB	M	17.60	13.42	0.001
	SD	4.43	4.77	
MCQ-CC	M	13.62	11.57	0.003
	SD	5.50	4.24	
MCQ-CSC	M	15.79	15.74	0.800
	SD	3.06	3.25	
MCQ-SPR	M	16.92	13.73	0.001
	SD	3.51	4.30	
CDI-2	M	26.44	14.49	0.001
	SD	9.94	9.01	

Note: DG—Depression group; WPDG—Without any psychiatric diagnosis group; *p*-value—probability value of T-Students Test; MCQ-PMB Positive Metacognitive Beliefs; MCQ-NMB Negative Metacognitive Beliefs; MCQ-CC—Cognitive confidence; MCQ-CSC Cognitive self-consciousness; MCQ-SPR—superstition, punishment, and responsibility; CDI-2 total score of depressive symptoms.

**Table 3:** Results of the multivariate logistic regression analysis on well-being.

Predictors	Odds Ratios	CI	Z	*p*
(Intercept)	0.00	0.00–0.05	−4.03	<0.001
FF	4.08	2.32–7.32	4.80	<0.001
DG	36.07	2.25–1012.34	2.34	0.019
MCQ-PMB	1.01	0.93–1.09	0.18	0.854
MCQ-NMB	1.06	0.98–1.14	1.45	0.148
MCQ-SPR	1.07	0.98–1.17	1.55	0.120
CDI-2	1.05	1.01–1.09	2.41	0.016
Age	1.18	1.01–1.39	2.03	0.042
FF × Group	33.92	4.04–683.52	2.76	0.006
Group × MCQ-PMB	0.62	0.43–0.82	−2.91	0.004
Observations	353			
R^2^	0.320			

Note: FF—Feeling of restriction of freedom; DG—Depression group; MCQ-PMB Positive Metacognitive Beliefs; MCQ-NMB Negative Metacognitive Beliefs; MCQ-SPR—superstition, punishment, and responsibility; CDI-2 total score of depressive symptoms; B = Unstandardized regression weight, Z = Z statistic score for B; OR = odds ratios; 95%CI.OR = 95% Confidence intervals for odds ratios; *p* = probability value.

**Table 4:** Results of the multivariate logistic regression of simple effects of interaction for the WPDG and DG on well-being.

	WPDG				DG			
Predictors	Odds Ratios	CI	Z	*p*	Odds Ratios	CI	Z	*p*
(Intercept)	0.00	0.00–0.04	−4.00	<0.001	0.12	0.00–833.25	−0.46	0.645
FF	4.05	2.29–7.29	4.75	<0.001	169.12	21.06–3823.00	4.00	<0.001
MCQ-PMB	1.01	0.93–1.10	0.23	0.815	0.65	0.44–0.85	−2.65	0.008
MCQ-NMB	1.06	0.98–1.15	1.36	0.173	1.00	0.78–1.27	0.03	0.979
MCQ-SPR	1.05	0.96–1.15	1.00	0.317	1.34	0.99–1.99	1.67	0.095
CDI-2	1.06	1.01–1.10	2.50	0.013	1.02	0.93–1.11	0.37	0.714
AGE	1.22	1.03–1.46	2.26	0.024	1.00	0.63–1.60	0.02	0.986
Observations	268				85			
R^2^	0.252				0.561			

Note: DG—Depression group; WPDG—Without any psychiatric diagnosis group; *p*-value—probability value of T-Students Test; MCQ-PMB Positive Metacognitive Beliefs; MCQ-NMB Negative Metacognitive Beliefs; MCQ-CC—Cognitive confidence; MCQ-CSC Cognitive self-consciousness; MCQ-SPR—superstition, punishment, and responsibility; CDI-2 total score of depressive symptoms; B = Unstandardized regression weight, Z = Z statistic score for B; OR = odds ratios; 95%CI.OR = 95% Confidence intervals for odds ratios; *p* = probability value.

**Table 5:** Results of the univariate logistic regression analysis on well-being.

	WPDG				DG			
Predictors	Odds Ratios	CI	Z	*p*	Odds Ratios	CI	Z	*p*
(Intercept)	-	-	-	-	-	-	-	-
FF	4.72	2.82–8.06	5.8	<0.001	20	6.50–72.67	4.92	<0.001
MCQ-PMB	1.06	0.99–1.14	1.65	0.100	0.88	0.77–1.00	−1.97	0.049
MCQ-NMB	1.17	1.10–1.24	5.2	<0.001	1.16	1.04–1.30	2.6	0.009
MCQ-SPR	1.16	1.09–1.25	4.44	<0.001	1.24	1.07–1.45	2.83	0.005
CDI-2	1.08	1.05–1.11	4.86	<0.001	1.06	1.01–1.12	2.35	0.019
AGE	1.32	1.13–1.54	3.49	<0.001	1.15	0.87–1.55	0.99	0.324

Note: DG—Depression group; WPDG—Without any psychiatric diagnosis group; *p*-value—probability value of T-Students Test; MCQ-PMB Positive Metacognitive Beliefs; MCQ-NMB Negative Metacognitive Beliefs; MCQ-CC—Cognitive confidence; MCQ-CSC Cognitive self-consciousness; MCQ-SPR—superstition, punishment, and responsibility; CDI-2 total score of depressive symptoms; Z = Z statistic score for B; OR = odds ratios; 95%CI.OR = 95% Confidence intervals for odds ratios; *p* = probability value.

## Data Availability

The data contained in this article have not been published in the repository but is available upon request.

## Appendix A

**Table A1:** Series of logistic regression models for all independent variables and interactions with Group in effect on well-being.

	Well-Being			
Predictors	Odds Ratios	CI	Z	*p*
A—Group × FF				
(Intercept)	0.48	0.32–0.71	−3.58	<0.001
Group	1.04	0.43–2.42	0.10	0.924
FF	4.72	2.82–8.06	5.80	<0.001
Group × FF	4.23	1.22–16.90	2.17	0.030
Observations	353			
R^2^	0.197			
B—Group × MCQ-NMB				
(Intercept)	0.82	0.41–1.64	−0.56	0.574
Group	1.04	0.24–4.37	0.05	0.961
MCQ-NMB	1.03	0.98–1.10	1.13	0.257
Group × MCQ-NMB	1.05	0.94–1.17	0.84	0.403
Observations	353			
R^2^	0.031			
C—Group × MCQ-CC				
(Intercept)	3.71	1.10−12.91	2.09	0.036
Group	0.34	0.02–5.41	−0.77	0.440
MCQ-CC	0.93	0.86–1.00	−1.85	0.065
Group × MCQ-CC	1.12	0.94–1.33	1.29	0.199
Observations	353			
R^2^	0.030			
D—Group × MCQ-SPR				
(Intercept)	0.15	0.06–0.38	−3.95	<0.001
Group	0.48	0.03–5.98	−0.56	0.576
MCQ-SPR	1.16	1.09–1.25	4.44	<0.001
Group × MCQ-SPR	1.06	0.91–1.26	0.75	0.455
Observations	353			
R^2^	0.105			
E—Group × CDI-2				
(Intercept)	0.41	0.25–0.66	−3.58	<0.001
Group	1.25	0.29–5.11	0.31	0.757
CDI-2	1.08	1.05–1.11	4.86	<0.001
Group × CDI-2	0.98	0.93–1.05	−0.51	0.609
Observations	353			
R^2^	0.109			
F—Group × Gender				
(Intercept)	1.86	0.89–3.90	1.65	0.098
Group	3.52	0.70–19.11	1.50	0.133
Gender	0.73	0.45–1.19	−1.25	0.213
Group × Gender	0.68	0.23–1.97	−0.72	0.474
Observations	353			
R^2^	0.029			
G—Group × The place of residence				
(Intercept)	1.21	0.67–2.18	0.63	0.529
Group	2.54	0.72–9.66	1.42	0.156
Place of residence	1.00	0.80–1.24	−0.03	0.975
Group × Place of residence	0.91	0.58–1.44	−0.39	0.697
Observations	353			
R^2^	0.020			
H—Group × age				
(Intercept)	0.02	0.00–0.18	−3.33	0.001
Group	15.40	0.10–2184.81	1.08	0.280
Age	1.32	1.13–1.54	3.49	<0.001
Group × Age	0.88	0.64–1.22	−0.79	0.429
Observations	353			
R^2^	0.058			

General note: DG—Depression group; WPDG—Without any psychiatric diagnosis group; *p*-value—*p*-probability value of T-Students Test; MCQ-PMB Positive Metacognitive Beliefs; MCQ-NMB Negative Metacognitive Beliefs; MCQ-CC—Cognitive confidence; MCQ-CSC Cognitive self-consciousness; MCQ-SPR—superstition, punishment, and responsibility; CDI-2- total score of depressive symptoms; Z = Z statistic; OR = odds ratios; 95%CI.OR = 95% Confidence intervals for odds ratios; *p* = probability value.

**Table A2:** Relation between the place of residence and Group classification before matching procedure.

	Group			
Predictors	Odds Ratios	CI	Z	*p*
(Intercept)	0.23	0.14−0.36	−6.11	<0.001
T50	1.33	0.67−2.64	0.83	0.407
C50	0.80	0.38−1.65	−0.60	0.550
Lc400	2.08	1.09−4.03	2.20	0.028
Observations	387			
R^2^	0.022			

Note: Z = Z statistic; OR = odds ratios; 95%CI.OR = 95% Confidence intervals for odds ratios; *p* = probability value.

**Table A3:** Relation between the place of residence and Group classification after matching procedure.

	Group			
Predictors	Odds Ratios	CI	Z	*p*
(Intercept)	0.30	0.18−0.48	−4.84	<0.001
T50	1.01	0.51−2.03	0.04	0.967
C150	0.71	0.34−1.49	−0.89	0.373
Lc400	1.58	0.82−3.10	1.36	0.175
Observations	353			
R^2^	0.015			

Note: Z = Z statistic; OR = odds ratios; 95%CI.OR = 95% Confidence intervals for odds ratios; *p* = probability value.
